# Epidemiology and outcomes of alpha‐1 antitrypsin deficiency in Sweden 2002–2020: A population‐based cohort study of 2286 individuals

**DOI:** 10.1111/joim.20058

**Published:** 2025-01-08

**Authors:** Staffan Wahlin, Linnea Widman, Hannes Hagström

**Affiliations:** ^1^ Department of Medicine Huddinge, Karolinska Institutet Stockholm Sweden; ^2^ Division of Hepatology, Department of Upper GI Diseases Karolinska University Hospital Stockholm Sweden

**Keywords:** incidence, liver cirrhosis, liver transplantation, lung transplantation, mortality, prevalence

## Abstract

**Objective:**

To estimate the incidence, prevalence, and outcomes of patients with diagnosed alpha‐1‐antitrypsin deficiency (AATD) in Sweden, 2002–2020.

**Study design and setting:**

The Swedish National Patient Registry was utilized to identify patients with a first diagnosis of AATD between 2002 and 2020. Each patient was matched with up to 10 comparators from the general population. AATD incidence and prevalence were estimated. Causes of death and rates of mortality, transplantation, lung disease, liver cirrhosis, and previous neonatal cholestasis were estimated.

**Results:**

The incidence rate of AATD was 1.83 (95% confidence interval [CI] 1.58–2.11) per 100,000 person‐years and the total prevalence was 21.04 (95%CI = 20.17–21.94) per 100,000 persons at the end of 2020. Mortality was 3.55 times higher (95%CI = 3.15–3.99) for patients with AATD. Rates of liver—(hazard ratio [HR] = 22.95, 95%CI = 12.61–41.75), lung—(HR = 12.09, 95%CI = 8.87–16.47), and cardiovascular (HR = 1.90, 95%CI = 1.45–2.90) related death were higher in patients with AATD. The cumulative incidence after 10 years of follow‐up was 1.69% (95%CI = 1.15–2.41) for liver transplantation and 4.14% (95%CI = 3.20–5.26) for lung transplantation. About 20% of patients were estimated to be alive without lung disease or liver cirrhosis 20 years after an AATD diagnosis. Neonatal cholestasis codes were found in 3.0% of AATD patients and 0.5% of comparators (odds ratio 6.28, 95%CI = 3.81–10.36).

**Conclusions:**

In this population‐based cohort study on AATD in Sweden, an increasing incidence was observed, and significantly higher rates of death from liver, lung, and cardiovascular causes compared to the general population were found. Only a minority of diagnosed AATD patients were estimated to be free of liver cirrhosis and lung disease after 20 years.

## Graphical abstract

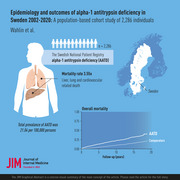

**Abbreviations**
AATD: alpha‐1‐antitrypsin deficiency
CI: confidence interval
HR: hazard ratio
IQR: interquartile range
NPR: national patient register
Pi*ZZ: homozygosity for the pathogenic Z variant in the SERRPINA1 gene (formerly Pi gene)
SES: socioeconomic statistics

## Introduction

Alpha‐1‐antitrypsin deficiency (AATD) is a rare genetic disorder caused by mutations in the SERPINA1 gene, which encodes alpha‐1‐antitrypsin (AAT) [1], a critical protein involved in protecting lung tissue from damage caused by neutrophil elastase. Although AATD lung disease is caused by AAT deficiency, AATD liver disease is caused by the aggregation of misfolded AAT in the liver. AAT deficiency or dysfunction is a prominent genetic cause of chronic pulmonary and liver diseases in adults, leading to significant morbidity and mortality [2].

The disease was described in 1962 by Laurell in Malmö, Sweden, where the Swedish AAT register was started in 1991. However, AATD is often unrecognized [3]. Approximately 20% of all Swedes with AATD are estimated to be registered [4]. It is unclear how many Swedish individuals are undiagnosed or diagnosed but not registered in the AAT database, which relies on active reporting from physicians caring for these patients.

The incidence, prevalence, natural history, and prognosis of this disease are incompletely known. AATD is considered an uncommon condition, with variable prevalence rates worldwide. The overall prevalence of AATD is estimated to range from 0 to 30 in 100,000 individuals in the general population [5, 6, 7, 8, 9]. There is significant regional and ethnic variation in the prevalence rates, and little is known about the incidence rates.

It is important to differentiate genetic AATD from clinically diagnosed AATD. Many studies have focused on the epidemiology of genetic variants in the SERPINA1 gene, but fewer have examined patients diagnosed with AATD. Individuals detected through screening have mortality rates similar to those of the general population [10]. Diagnosed AATD patients likely represent the AATD patient populations observed in clinical medicine. Because clinically diagnosed AATD is rare, its frequency and outcome may be best studied in population‐based healthcare databases.

Our primary objectives were to describe the demographic characteristics of patients with newly diagnosed AATD in Sweden from 2002 to 2020, to estimate the incidence and prevalence of diagnosed AATD as of December 31, 2020, and to provide risk estimates for outcomes including overall mortality and lung and liver transplantation.

The secondary objectives were to estimate the proportions of individuals diagnosed at a pulmonary or gastroenterology department, as a proxy for the first disease manifestation; to provide risk estimates for incident nonfatal lung disease and cirrhosis; to estimate proportions of patients with newly diagnosed AATD with comorbidities at baseline, including lung disease and cirrhosis; and to investigate whether neonatal cholestasis, sometimes the presenting symptom, was more common among individuals later diagnosed with AATD.

## Methods

We utilized the DELIVER cohort [11], containing data from, among other sources, the Swedish National Patient Registry (NPR) to identify all individuals diagnosed with AATD in Sweden from January 1, 1987 to December 31, 2020. Since 2001, the NPR has contained both administrative coding from discharges from any hospital and data on all outpatient visits in specialized care in Sweden. The NPR's positive predictive value for most chronic diseases ranges between 85% and 95% [12] and is over 90% for diagnoses associated with cirrhosis, including hepatocellular carcinoma [13].

The presence of AATD was defined using the International Classification of Disease (ICD) codes E88.0A and E88.0B (ICD‐10) or 277G (ICD‐9). The start of follow‐up (study baseline) was the first date of ICD coding for AATD per patient in the NPR. Each patient with AATD was compared with up to 10 reference individuals from the general population, matched for age, sex, calendar year of AATD diagnosis, and municipality at baseline. These comparators were identified through cross‐linking with the Total Population Registry, which contains data on the dates of birth, migration, death, and other demographic parameters. Matching was performed with direct matching using the central Swedish authority Statistics Sweden on the date of the first diagnosis of any liver disease.

**Fig. 1:**
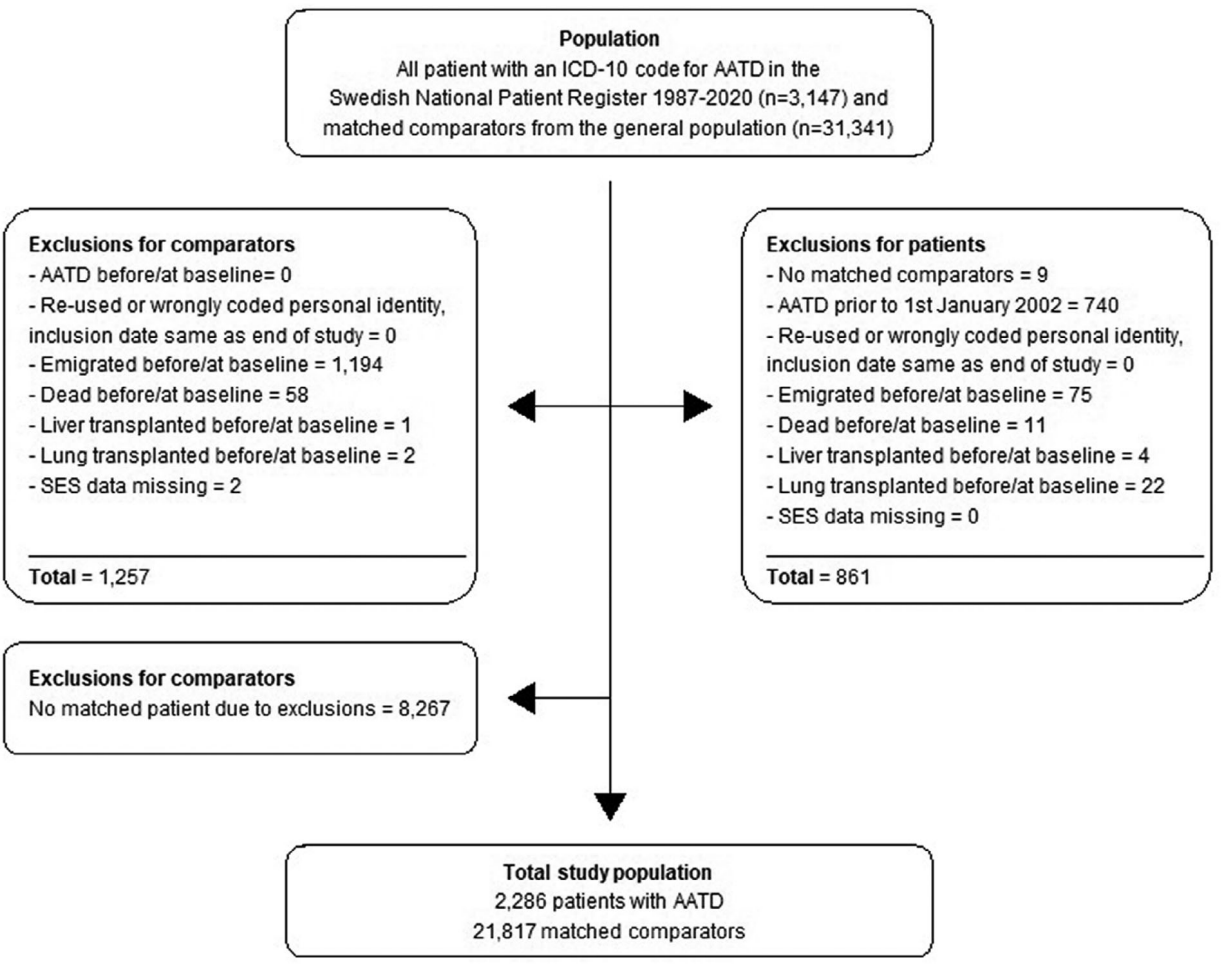
Flowchart of study inclusion and exclusion criteria. AATD, alpha‐1‐antitrypsin disease; SES, socioeconomic statistics including country of birth.

We excluded individuals who met any of the following criteria at baseline: reused personal identity numbers, a diagnosis of AATD between 1987 and the end of 2001 (this was done to exclude those with prevalent AATD at the start of the study period to ensure the identification of only newly diagnosed patients, assuming that patients with prevalent AATD would have at least one visit in specialized care), formally emigrated from Sweden, dead at baseline, previous liver or lung transplant, or other administrative reasons (Fig. 1).

The primary outcome was overall mortality obtained from the National Causes of Death Registry. The Causes of Death Registry comprises data on all deaths in Sweden using a two‐step process. First, a death certificate in which a physician confirms death is sent to the Swedish tax office. This certificate must be completed before the burial is authorized. The second step entailed a report on the cause of death filled in by a physician and sent to the National Board of Health and Welfare [14].

Secondary outcomes included cause‐specific mortality and cumulative probability of lung disease, liver cirrhosis, and death without liver transplantation, as well as liver transplantation, liver‐related death, lung‐related death, cardiovascular‐related death, primary liver cancer (PLC), and non‐hepatic cancers. Data on previous neonatal cholestasis (defined by ICD‐8, 9, and 10 codes), liver transplantation, and lung transplantation were obtained from the NPR based on ICD‐codes. The outcomes were defined using ICD‐10 codes (where not otherwise specified) as shown in detail in Table S1, together with comorbidities used. The secondary outcomes were sourced from the Swedish inpatient and outpatient registries, and the cause of death register. Follow‐up was until death, emigration, or December 31, 2020, whichever occurred first.

### Statistical methods

Continuous data are presented as medians and 25th and 75th percentiles (interquartile range [IQR]), and categorical variables as frequencies and percentages. The Wilcoxon rank‐sum test was used to investigate differences between groups for continuous variables and Pearson's *χ* ^2^ test for categorical variables. We calculated incidence rates per 100,000 person‐years of follow‐up. Cumulative incidences for overall mortality were estimated using the Kaplan–Meier method. Cox regression was used for comparing overall mortality within the matched strata of AATD patients and reference individuals, thereby adjusting for the matching factors (age, sex, municipality, and year of diagnosis). The incidence of diagnosed AATD was calculated by dividing all new cases with the Swedish total population alive on December 31 for the years 2002–2020. Total prevalence was calculated for each year using all patients still alive at the end of the year with an existing AATD ICD‐9/10 code. Total population numbers for each year were retrieved from Statistics Sweden. Both the incidence and prevalence estimations included individuals we excluded for our specific study (Fig. 1). 95% confidence intervals (CIs) for both incidence and prevalence were calculated with the Clopper–Pearson exact CI (*R: PropCIs::exactci*) for proportions. The trends over time were tested with linear regression.

For the secondary outcomes, we used cumulative incidence from the Aalen–Johansen method (STATA: *stcompet*) to compare the rates and risks of outcomes in patients and reference individuals. In these analyses, non‐outcome mortality was considered a competing‐risk event (e.g., when investigating liver‐related mortality, non‐liver mortality was considered a competing event).

Patients with AATD may develop different phenotypes, for example, cirrhosis but no lung disease. To visualize the probabilities for different states, we performed a multistate survival analysis (*R: survminer::ggcompetingrisks*) to estimate the following states: (1) alive without any lung disease or cirrhosis, (2) alive with lung disease, (3) alive with cirrhosis, (4) alive with both lung disease and cirrhosis, (5) dead with lung disease, (6) dead with cirrhosis, (7) dead with both lung disease and cirrhosis, and (8) dead without any lung disease or cirrhosis. All analyses were performed using STATA version 16.1 and R version 4.3.1. (ICD‐codes are described in Table S1).

## Results

We identified 3147 individuals with a record of an AATD diagnosis in Sweden between 1987 and 2020. After exclusions, 2286 individuals diagnosed between 2002 and 2020 remained (Fig. 1). The median age at diagnosis was 51 years; 46% were men, 97% were born in the Nordic countries (Sweden, Norway, Denmark, Finland, or Iceland), 44% had a pulmonary disease diagnosis at or before baseline, and 5% had a liver disease diagnosis at or before baseline. The AATD diagnosis was first established mainly in a pulmonary clinic (39%), an internal medicine clinic (32%), or a pediatric clinic (13%) (Table 1).

**Table 1:** Baseline characteristics of individuals with alpha‐1‐antitrypsin disease (AATD) and matched general population reference individuals.

	AATD (*N* = 2286)	Reference individuals (*N* = 21,817)	*p*
Follow‐up (years, median, IQR)	5.95 (2.46–10.10)	6.86 (2.93–11.04)	<0.001
Sex, men, *n* (%)	1054 (46.1)	10,049 (46.1)	0.97
Age at inclusion (years, median, IQR)	51.00 (34.00–63.00)	51.00 (33.00–63.00)	0.69
Period of inclusion, year, *n* (%)			0.99
2002–2005	230 (10.1)	2222 (10.2)	
2006–2010	548 (24.0)	5263 (24.1)	
2011–2015	699 (30.6)	6665 (30.5)	
2016–2020	809 (35.4)	7667 (35.1)	
Country of birth, *n* (%)			<0.001
Nordic	2221 (97.2)	18,951 (86.9)	
Other	6.5 (2.8)	2866 (13.1)	
Clinic making the first diagnosis, *n* (%)			
Pulmonary	893 (39.1)	NA	
Gastroenterology	54a (2.4)	NA	
Paediatric	304 (13.3)	NA	
Internal medicine	741 (32.4)	NA	
Other	292 (12.8)	NA	
Missing	2 (0.1)	100%	
Comorbidity at or before baseline, *n* (%)			
Lung disease	1016 (44.4)	1174 (5.4)	<0.001
Liver cirrhosis	90 (3.9)	0 (0)	<0.001
Neonatal cholestasis	28 (1.2)	44 (0.2)	<0.001
Primary liver cancer	11 (0.5)	2 (0.0)	<0.001
Extra‐hepatic cancer	265 (11.6)	1907 (8.7)	<0.001
Any previous liver disease	107 (4.7)	16 (0.1)	<0.001
Deceased during study period, *n* (%)	404 (17.7)	1651 (7.6)	<0.001

Abbreviation: IQR, inter‐quartile range.

**Fig. 2:**
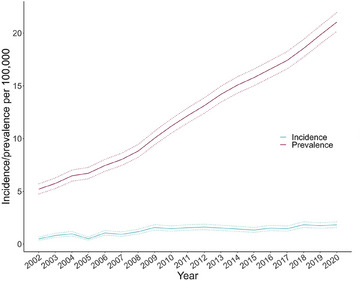
Incidence and prevalence of diagnosed alpha‐1‐antitrypsin disease (AATD) in Sweden 2002–2020. Both increased significantly during the study period (p < 0.001).

The incidence and prevalence of AATD significantly increased during the study period (*p* < 0.001) (Fig. 2). The incidence rate was 1.83 (95%CI 1.58–2.11) per 100,000 person‐years, and the prevalence of AATD was 21.04 (95%CI 20.17–21.94) per 100,000 persons at the end of study.

**Fig. 3:**
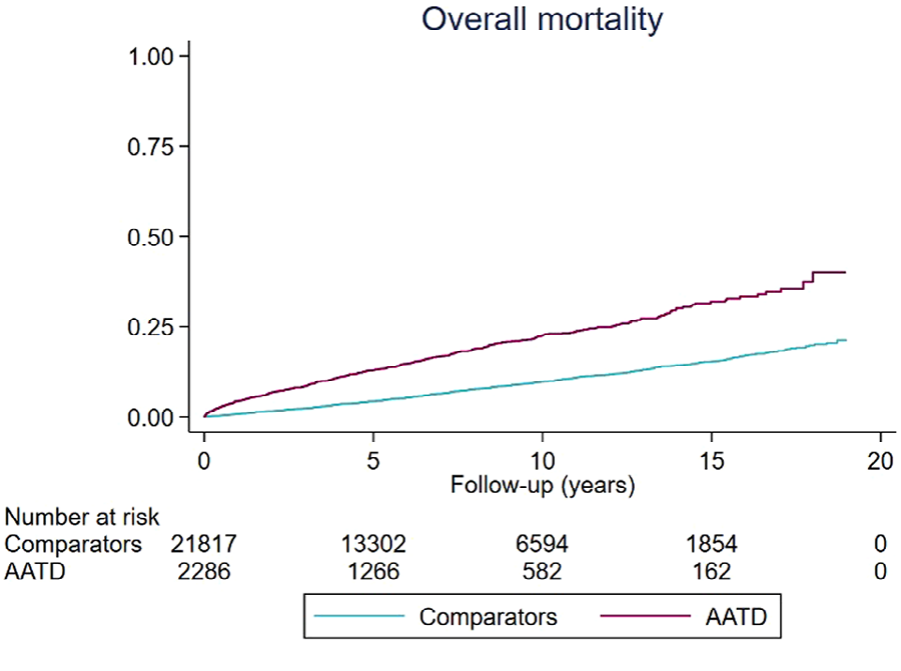
Cumulative all‐cause mortality among individuals diagnosed with alpha‐1‐antitrypsin disease (AATD) and reference individuals.

The 2286 included patients with AATD were compared to 21,817 matched general population reference individuals. During a median follow‐up of 5.91 years (IQR 2.46–10.10), there were 404 deaths (17.7%) in the AATD group. During a median follow‐up of 6.86 years (IQR 2.93–11.04), there were 1651 deaths (7.6%) in the reference group. The overall mortality rate per 1000 person‐years was 26.5 (95%CI 24.0–29.2) among patients with AATD and 10.3 (95%CI 9.8–10.8) among reference individuals, translating into a hazard ratio (HR) of 3.6 (95%CI 3.2–4.0) for overall mortality (Table 2). Kaplan–Meier estimates for cumulative overall mortality in patients with AATD were 4.4% (3.5–5.3) over 1 year, 13.0% (11.5–14.5) over 5 years, and 22.7% (20.5–25.0) over 10 years (Table 3, Fig. 3).

**Table 2:** Overall mortality and secondary outcomes in individuals with alpha‐1‐antitrypsin disease (AATD) and reference individuals.

	AATD (*N* = 2286)		Reference individuals (*N* = 21,817)			
	Outcome *N* (%)	Outcome per 1000 person‐years (95%CI)	Outcome *N* (%)	Outcome per 1000 person‐years (95%CI)	*HR (95%CI)*	*p*
Overall mortality	404 (17.7)	26.50 (24.03–29.21)	1651 (7.6)	10.33 (9.85–10.85)	3.55 (3.15–3.99)	<0.001
Cause‐specific mortality						
Liver‐specific	38 (1.7)	2.49 (1.81–3.43)	26 (0.1)	0.16 (0.11–0.24)	22.95 (12.61–41.75)	<0.001
Lung‐specific	99 (4.3)	6.49 (5.33–7.91)	117 (0.5)	0.73 (0.61–0.88)	12.09 (8.87–16.47)	<0.001
CVD mortality	65 (2.8)	4.26 (3.34–5.44)	546 (2.5)	3.42 (3.14–3.72)	1.90 (1.45–2.49)	<0.001
Malignancies other than PLC	50 (2.2)	3.27 (2.49–4.33)	459 (2.1)	2.87 (2.62–3.15)	1.35 (1.00–1.82)	0.051
Other	66 (2.9)	4.32 (3.40–5.51)	503 (2.3)	3.15 (2.89–3.44)	1.96 (1.50–2.57)	<0.001
Other outcomes						
Liver transplantation^a^	32 (1.4)	2.12 (1.50–3.01)	1	–	–	–
Lung transplantation	73 (3.2)	4.90 (3.89–6.16)	0	–	–	–

Abbreviations: CI, confidence interval; HR, hazard ration; CVD, cardiovascular disease; PLC, primary liver cancer.

^a^ A single liver transplantation among reference individuals precludes meaningful analysis of HR.

**Table 3:** Cumulative overall mortality and cumulative incidence of outcomes after 1, 5, and 10 years in individuals with alpha‐1‐antitrypsin disease (AATD) and reference individuals.

	AATD			Reference individuals		
	1 year	5 years	10 years	1 year	5 years	10 years
Overall mortality	4.40 (3.50–5.31)	12.95 (11.47–14.52)	22.68 (20.49–24.95)	0.74 (0.63–0.87)	4.30 (4.00–4.61)	9.71 (9.19–10.24)
Cause‐specific mortality						
Liver‐specific	0.87 (0.54–1.33)	1.42 (0.97–2.01)	2.12 (1.47–2.95)	0.01 (0.00–0.04)	0.08 (0.04–0.13)	0.15 (0.10–0.24)
Lung‐specific	1.04 (0.68–1.53)	3.14 (2.41–4.01)	5.99 (4.81–7.34)	0.63 (0.04–0.11)	0.30 (0.22–0.39)	0.70 (0.57–0.86)
CVD mortality	0.91 (0.57–1.38)	1.90 (1.35–2.58)	3.68 (2.78–4.77)	0.26 (0.20–0.34)	1.43 (1.26–1.61)	3.18 (2.88–3.49)
Malignancies other than PLC	0.50 (0.27–0.88)	1.80 (1.27–2.49)	2.62 (1.91–3.50)	0.24 (0.18–0.31)	1.35 (1.18–1.53)	2.75 (2.47–3.05)
Other	0.50 (0.27–0.88)	2.15 (1.55–2.89)	3.65 (2.76–4.71)	0.18 (0.13–0.24)	1.17 (1.01–1.34)	2.95 (2.66–3.26)
Other outcomes						
Liver transplantation^a^	0.83 (0.51–1.29)	1.31 (0.88–1.88)	1.69 (1.15–2.41)	(*N* = 1)	(*N* = 1)	(*N* = 1)
Lung transplantation	0.87 (0.53–3.25)	2.47 (1.83–3.25)	4.14 (3.20–5.26)	0	0	0

*Note*: Numbers shown as percentages with 95% confidence intervals.

Abbreviations: CIs, confidence intervals; CVD, cardiovascular disease, PLC, primary liver cancer.

^a^ A single liver transplantation among reference individuals precludes meaningful statistical analysis.

Mortality was similar between men and women. In the subgroup of AATD patients with a liver (*n* = 107) or lung (*n* = 1016) diagnosis at or before baseline, the HRs for mortality compared to their matched reference individuals were 8.13 for liver (95%CI 4.76–13.83) and 4.71 for lung disease (95%CI 4.10–5.40), significantly higher than the corresponding HRs among AATD patients without liver or lung disease (HR 3.41 (95%CI 3.02–3.84) and HR 1.77 (95%CI 1.39–2.26) (*p *< 0.001 for all) (Table 4).

**Table 4:** Cumulative overall mortality depending on sex and baseline morbidity.

	AATD (*N* = 2286)			Reference individuals (*N* = 21,817)				
	Exposed in subgroup N	Outcome *N* (%)	Incidence rate	Exposed in subgroup *N*	Outcome *N* (%)	Incidence rate	*HR (95%CI)*	*p*
Men	1054	213 (20.2)	30.14 (26.35–34.47)	10,049	821 (8.2)	10.95 (10.22–11.72)	3.89 (3.30–4.59)	<0.001
Women	1232	191 (15.5)	23.35 (20.26–26.91)	11,768	830 (7.1)	9.79 (9.15–10.48)	3.23 (2.73–3.82)	<0.001
With liver disease at baseline	107	27 (25.2)	41.45 (2*8.42–60.44*)	986	71 (7.2)	9.96 (7.89–12.56)	8.13 (4.78–13.83)	<0.001
Without liver disease at baseline	2179	377 (17.3)	25.83 (23.35–28.57)	20,831	377 (17.3)	10.35 (9.86–10.88)	3.41 (3.02–3.84)	<0.001
With lung disease at baseline	1016	323 (31.8)	50.65 (45.42–56.49)	9690	1127 (11.6)	15.40 (14.52–16.32	4.71 (4.10–5.40	<0.001
Without lung disease at baseline	1270	81 (6.4)	9.13 (7.35–11.35)	12,127	524 (4.3)	6.06 (5.56–6.60)	1.77 (1.39–2.26)	<0.001

**Fig. 4:**
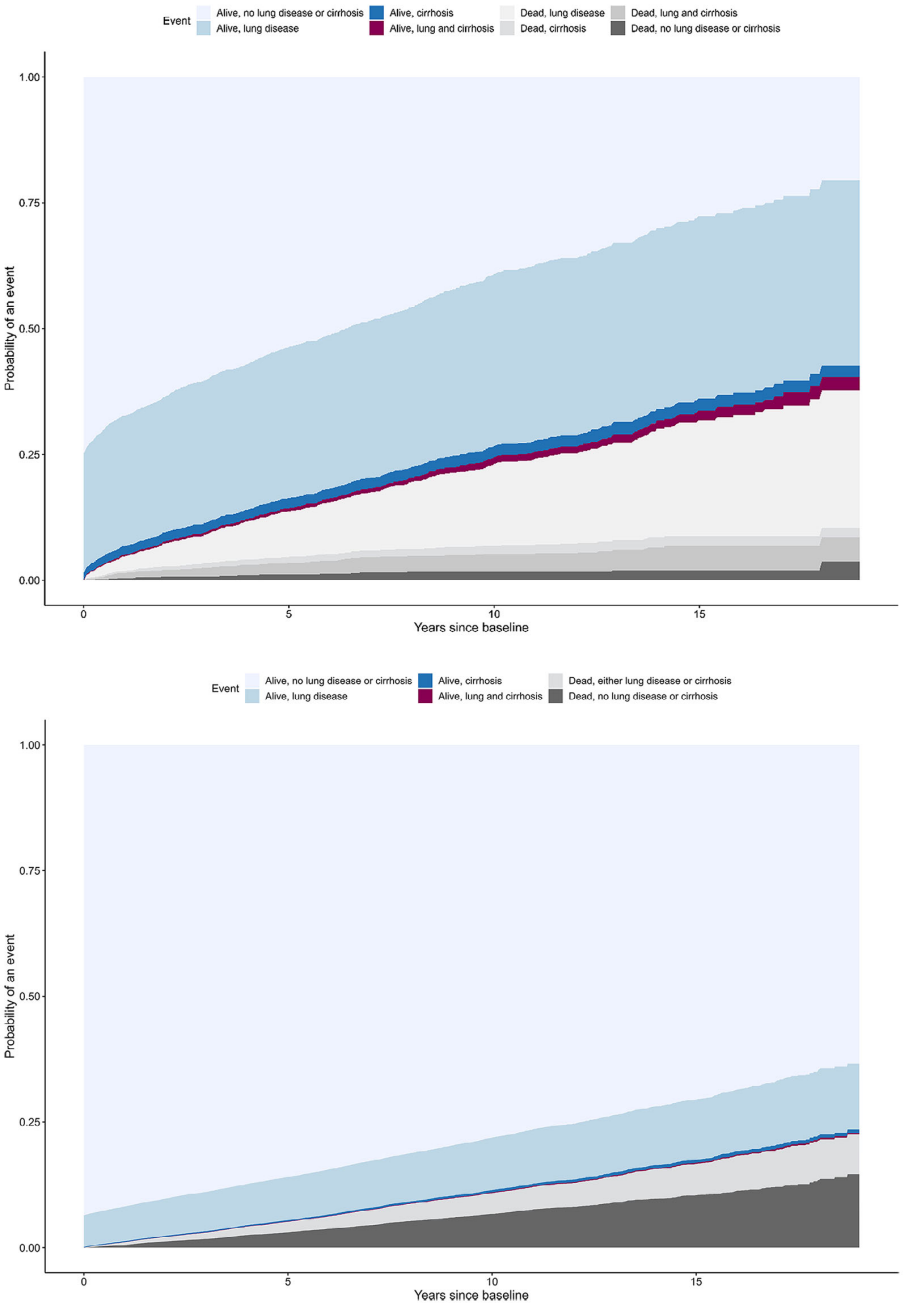
The cumulative probability of lung disease, liver cirrhosis, and death without liver transplantation as competing‐risk events in a multistate analysis among (a) individuals with alpha‐1‐antitrypsin disease (AATD) and (b) matched reference individuals.

Among patients with AATD, 32 (1.4%) underwent liver transplantation. There was only one case of liver transplantation among the comparators. For the composite endpoint of death or liver transplantation, the cumulative incidences in patients with AATD were 4.4% over 1 year, 13.0% over 5 years, and 22.7% over 10 years (Table 3, Fig. 3). Figure 4 shows the cumulative incidence of lung disease, liver cirrhosis, and death without liver transplantation in a multistate analysis among individuals with AATD (Fig. 4a) and reference individuals (Fig. 4b).

We observed higher HRs in the AATD group for death from lung and liver‐related causes and cardiovascular disease (CVD), but not for non‐liver malignancies (Table 2).

Among 941 (41.2%) AATD patients born in 1967 or later, meaning their diagnoses could be captured in the used registries during the full lifespan (ICD8–10 eras), 28 (3.0%) had a diagnosis of neonatal cholestatic liver disease in the first year of life, compared with 44 (0.5%) of the comparators (OR 6.28, 95%CI = 3.81–10.4).

## Discussion

In this first nationwide Swedish study based on AATD ICD‐codes in the national patient registers, individuals diagnosed with AATD exhibited an almost fourfold increased rate of death compared with matched comparators from the general population. The cumulative mortality over 10 years after a diagnosis of AATD was 23% compared to 10% in the reference population, at a median age at first diagnosis of 51 years. Thirty‐two (1.4%) patients with AATD underwent liver transplantation, and 73 (3.2%) underwent lung transplantation. About 20.5% were estimated to be alive without lung disease or liver cirrhosis 18.9 years after AATD diagnosis (Fig. 4a).

The incidence of diagnosed AATD in Sweden was 1.83, and the prevalence was 21.04 per 100,000 at the end of 2020 (Fig. 2). A recent register‐based study in Denmark similarly found an incidence of 1.59/100,000 in 2015–2018 and a total prevalence of 21.4 per 100,000 in a comparable analysis [9].

A limited number of studies have reported long‐term mortality rates in AATD. Study results are heavily dependent on the characteristics of the study cohort, on whether the AATD diagnosis is based on genetic screening or patients with clinical lung or liver disease [10, 15, 16, 17]. Screening for pathogenic SERPINA1 genotype Pi*ZZ variant among Swedish infants yielded a frequency of 1/1550 (65/100,000) [18]. Less than 25% of individuals with genotypic AATD are estimated to be identified and diagnosed with AATD [4, 19]. Our study, which also includes clinically diagnosed patients with AATD, suggests that approximately 30% are identified and diagnosed in Sweden. Our results should be interpreted with that in mind.

We found a predominance for lung disease (Fig. 4a) and lung‐related causes of death (Table 2) among patients with AATD. Predominance of lung disease is well known in AATD [20] but liver disease has a major impact on mortality. The risk of mortality was especially high if liver disease was present at or before AATD diagnosis (HR 8.13). Individuals with the SERPINA1 Pi*ZZ genotype in the Swedish AATD register who were followed until 43–45 years of age had a similar life expectancy as the Swedish general population [21]. Most deaths in that study were liver‐associated deaths during childhood. A study from 2008 estimated that causes of death in Pi*ZZ AATD were respiratory in 40%, hepatic in 25%, and other in 35%, based on 93 deaths [16]. The corresponding numbers in our study were as follows: respiratory in 31%, hepatic in 12%, and other in 41% among 404 deaths, of which half were due to CVD (Table 2). Previous studies have reported both higher and lower risk for CVD in individuals with AATD [17, 22, 23, 24].

A mere 1.2% of individuals with AATD born in 1967 or later had a registered code for neonatal cholestasis during their first year of life, although this was sixfold more common than among comparators. Neonatal cholestasis is one of the major clinical presentations of AATD and constitutes a risk of early progression to severe liver disease and liver transplantation [25, 26]. We did not, due to few patients with recorded neonatal cholestasis, analyze if such risks could be confirmed in our cohort.

The strengths of our study include the nationwide coverage of the registries used to identify patients with AATD, mitigating selection bias. Our study thereby provides a comprehensive picture of long‐term outcomes in a large cohort of patients with AATD in real‐world practice in Sweden. The inclusion of patients only from the modern era (2002–2020) and comparison to matched population reference individuals are additional strengths. The national, population‐based registers used for ascertaining exposure and outcome status are validated and are sources of high‐quality data, allowing for ascertaining long‐term outcomes and virtually no loss to follow‐up [12, 14, 27]. The large size of the cohort means that risk estimates could be more precise than previous smaller studies.

We acknowledge some limitations, most of which result from a lack of granular data on clinical characteristics. Morbidity and mortality depend on genotype [15], and our data set included no data to confirm that most individuals with AATD had the PI*ZZ genotype in *SERPINA1*. Some included individuals may carry the less severe PiSZ genotype. It is, however, a reasonable assumption that an AATD ICD‐code is registered only for individuals with low serum AAT levels (<11 µM or <0.6 g/L) in a clinically diagnosed cohort. We did not have access to data in the Swedish AAT register.

We had no information on AAT serum levels, smoking, or other risk factors for registered outcomes. Such information is not available in the registries used for analyses containing administrative data. The ICD‐10 code for AATD is not formally validated, and there remains a risk of misclassification. Our analyses of the presence and severity of liver disease are incomplete. We used ICD‐codes to exclude individuals with preexisting liver diagnoses before the AATD diagnosis, but only ICD‐codes for liver cirrhosis and PLC for outcome analyses, lacking data on fibrosis stage or other characteristics (see Table S1 for ICD‐codes). The presence of non‐cirrhotic liver disease during follow‐up could therefore not be accurately assessed. Likewise, we only had access to ICD‐codes to define lung disease broadly and no data on pulmonary function.

To address the gaps in knowledge about the natural history and outcome of AATD, large AAT registers have been started. The Alpha One International Registry (AIR) included 14 European and some non‐European countries [28]. AIR successfully collected baseline data, but longitudinal data were never collected due to limited funding. In a new effort to collect longitudinal data on AATD, the new pan‐European EARCO register was started in 2019 [29]. EARCO has not yet reported any longitudinal data. Our nationwide study based on AATD ICD‐codes in the NPR aims to be an addition to the limited available data on natural history and outcome of diagnosed AATD and may be used to inform patients on risks for adverse outcomes.

In conclusion, in a nationwide population‐based cohort study, the incidence and prevalence of diagnosed AATD in Sweden were 1.83 and 21.04 per 100,000 at the end of 2020. Patients with AATD had an almost fourfold increased rate of death compared with matched individuals from the general population. The risks for needing a liver or lung transplant and death from lung‐, liver‐, and CVD were especially elevated.

## Author contributions

*Study conception and design*: Staffan Wahlin, Hannes Hagström. *Acquisition of data*: Hannes Hagström. *Statistical analysis*: Linnea Widman. *Analysis and interpretation of data*: All. *Drafting of manuscript*: Staffan Wahlin. *Critical revision*: All. *Guarantors of the article*: Staffan Wahlin and Hannes Hagström. All authors have approved the final version of the article, including the authorship list. *Writing assistance*: None.

## Conflicts of interest statement

The authors declare no conflicts of interest.

## Funding information

No specific funding was obtained for this study.

## Ethics statement

The study was approved by the Regional Ethics Review Board in Stockholm (dnr 2017/1019‐31/1).

## Patients consent statement

As this study included analyses of de‐identified data, written consent from the participants was not required.

## Supporting information




**Supplementary Table 1**: ICD‐codes used for defining AATD, exclusion criteria, covariates and outcomes used for different analyses in the study.

## Data Availability

The data that support the findings of this study are available from the corresponding author upon reasonable request.
