# Supplementary material for: Epidemiology and outcomes of alpha‐1 antitrypsin deficiency in Sweden 2002–2020: A population‐based cohort study of 2286 individuals
Source: J Intern Med. 2025 Jan 8;297(3):300–11. doi: 10.1111/joim.20058 (PMC11846075; doi:10.1111/joim.20058)
Supplement: Supplementary file 1 — Supplementary Table 1: ICD‐codes used for defining AATD, exclusion criteria, covariates and outcomes used for different analyses in the study. [file JOIM-297-300-s001.docx]

**Supplementary Table 1.**

ICD-codes used for defining AATD, exclusion criteria, covariates and outcomes used for different analyses in the study.

| **Definitions** | **ICD-10** | **ICD-9** |
| --- | --- | --- |
| AATD | E88.0A, E88.0B | 277G |
| Previous liver disease | B16-B19, E83.0B, E83.1, I82.0, K71, K74.3-K74.5, K75.4, K75.8, K76.0, K76.5, K83.0, Z94.4  Procedure codes: DJ005, DJ006, JJC00, JJC10, JJC20, JJC30, JJC40 | 070, 275A-275B, 453A, 571E, 571G, 571W, 576B, V42H  Procedure codes: 5200 |
| Previous lung disease | J40-J47, J80, J84 | 490-494, 515, 516, 518F |
| Neonatal cholestasis | K70-K74, P57, P58, P78 | ICD-9: 774B–774X  ICD-8: 570-577, 774, 785 |
| Liver cirrhosis | I85.0, I85.9, I98.2-I98.3, K70.3, K72.1, K72.9, K74.6, K76.6-K76.7, R18 | 456A-456C, 571C, 571F, 572C-572E, 572W, 789F |
| Liver transplantation | Z94.4  Procedure codes: DJ005, DJ006, JJC00, JJC10, JJC20, JJC30, JJC40 | V42H  Procedure codes: 5200 |
| Lung transplantation | Z942  Procedure codes: GDG00, DG03, GDG10, GDG13, GDG30, DG96 | V42G,  Procedure codes: 3350-3352 |
| Liver-related cause of death | C22, E88.0, I85.0, I85.9, I98.2, I98.3, K70-K77, Z94.4 |  |
| Lung-related cause of death | J00-J99, Z94.2 |  |
| CVD-related cause of death | I00-I99 |  |
| Other causes of death | Any code not corresponding to the four above definitions |  |
| Non-hepatic cancers | Any C code, excluding C22 | 140-239 except 155 |
| Primary liver cancer | C22 | 155 |
